# Ferroelectric Domain Wall p–n Junctions

**DOI:** 10.1021/acs.nanolett.3c02966

**Published:** 2023-11-10

**Authors:** Jesi R. Maguire, Conor J. McCluskey, Kristina M. Holsgrove, Ahmet Suna, Amit Kumar, Raymond G. P. McQuaid, J. Marty Gregg

**Affiliations:** † School of Mathematics and Physics, Queen’s University Belfast, Belfast BT7 1NN, U.K.

**Keywords:** ferroelectrics, domains, domain walls, p−n junctions, domain-wall electronics

## Abstract

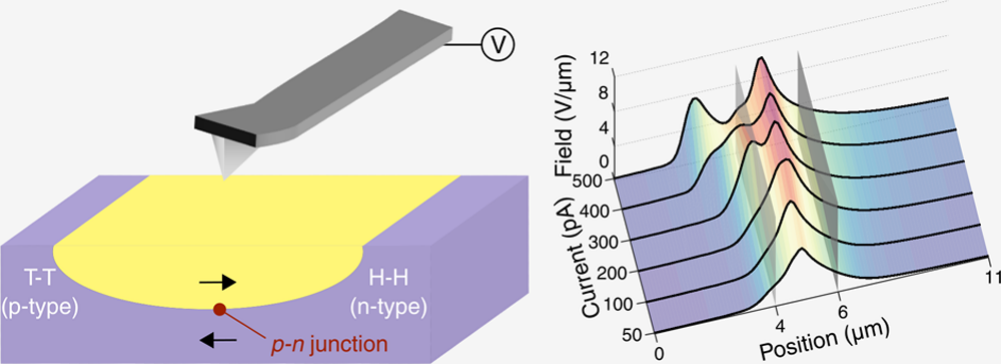

We have used high-voltage Kelvin probe force microscopy to map the spatial distribution of electrical potential, dropped along curved current-carrying conducting domain walls, in x-cut single-crystal ferroelectric lithium niobate thin films. We find that *in-operando* potential profiles and extracted electric fields, associated with *p–n* junctions contained within the walls, can be fully rationalized through expected variations in wall resistivity alone. There is no need to invoke additional physics (carrier depletion zones and space-charge fields) normally associated with extrinsically doped semiconductor *p–n* junctions. Indeed, we argue that this should not even be expected, as inherent Fermi level differences between *p* and *n* regions, at the core of conventional *p–n* junction behavior, cannot occur in domain walls that are surrounded by a common matrix. This is important for domain-wall nanoelectronics, as such in-wall junctions will neither act as diodes nor facilitate transistors in the same way as extrinsic semiconducting systems do.

By definition, a ferroelectric material has a spontaneous polarization, which can be reoriented by an externally applied electric field.^1^ Superficially, such a requirement for “switching” is incommensurate with significant electrical conductivity, and it is true that most ferroelectrics are reasonably insulating. However, domain walls within ferroelectrics (interface structures that separate regions of uniformly oriented dipoles) can be very notably different;^2^ under ambient conditions, wall conductivity can be more than 13 orders of magnitude greater than bulk,^2,3^ while, under cryogenic conditions, wall superconductivity can even be seen.^4^

The physics responsible for generating domain-wall conduction is still under debate and the reality is that details vary from one system to another.^5^ In general, though, domain walls across which there are discontinuities in polarization (so-called “charged” walls) are most reliably seen to conduct.^2,3,6−16^ In such cases, conductivity scales with the magnitude of the divergence in polarization at the wall, which can often be “tuned” by changing wall orientation.^2,6,8,11,17^ Carrier type can also be tuned: walls which support head-to-head polar discontinuities accumulate negative screening charges and show *n*-type transport behavior,^18^ while tail-to-tail walls accumulate positive screening charge and are found to be *p*-type.^13,18−20^

Junctions between head-to-head and tail-to-tail walls (in principle, *p–n* junctions) are more commonly found than one might imagine, particularly in improper ferroelectric systems, such as the boracites^13,14^ and the rare-earth manganites.^8,15,19−21^ They can emerge either through the intersection of two distinct domain walls with different crystallographic orientations or through single wall orientational meandering.^21^ Junctions are certainly not completely restricted to improper ferroelectrics. In lithium niobate (LNO, a uniaxial proper ferroelectric), Jiang and co-workers^22,23^ have consistently seen that complete switching of their capacitor devices produces a single curved domain wall (which has been described as the hull of a boat). Close to the positive electrode, this wall is always tail-to-tail, while close to the negative electrode it is head-to-head; hence, within the interelectrode gap, the “polarity” of the wall must reverse and a *p–n* junction must form, although this has not been explicitly noted in the literature to date.

While many 2D conducting systems exist, ferroelectric domain walls are particularly exciting because they can be moved around within the insulating ferroelectric matrix as domains expand and contract under the influence of external fields. Indeed, during switching, domains and domain walls may be created and destroyed; domain-wall-based conducting conduits are hence inherently mobile and ephemeral in nature, and this has prompted their use in completely new forms of transient devices and electronic circuitry.^3,22−27^ In principle, mobile and ephemeral *p–n* junctions could be extremely important, in this context, and it is hence surprising that greater efforts to characterize their properties have not been made. The single most relevant report to date concerns the functional characterization of a domain-wall *p–i–n* junction.^18^ Dramatic diode-like characteristics were seen in this study. However, the form of the junction was rather contrived (it was essentially a capacitor structure with n*-* and p*-*type domain walls acting as parallel-plate electrodes, separated by a 8 μm thick slab of monodomain LNO). It is not, therefore, clear that the properties observed were related specifically to the change in carrier type. Perhaps other asymmetries involved (such as the sense of polarization in the capacitor structure) might have been dominant in determining the observed current–voltage response.

Against this background, we herein report on the in-operando characterization of domain walls containing *p–n* junctions that form in single-crystal ion-sliced x-cut LNO, after bias-induced switching, using coplanar surface electrodes. We have used high-voltage Kelvin probe force microscopy (KPFM) to map the potential profile along the LNO surface, which acts electrically in parallel with the current-carrying domain wall. We thereby derived the distribution of the electric field associated with conduction. In general, three field peaks were seen: one that we suspect is related to a Schottky barrier at the high-potential contact between the surface electrode and the domain wall; one that seems to be related to the fringing fields at the electrode edge; and one in the middle of the interelectrode gap, which we presume to be associated with the domain-wall *p–n* junction present at that point. We directly determined the morphology of the domain wall (through both tomographic piezoresponse force microscopy (TPFM) and cross-sectional transmission electron microscopy (TEM)) and found it to approximate to the surface of a column with its long axis parallel to the film surface and with a hemielliptical cross section. By modeling the magnitude of the polarization discontinuity, as a function of position along the hemielliptical section, we determined the expected local variation in wall resistivity and found that this maps well to the field peak associated with the in-wall *p–n* junction. No additional physics relating to charge depletion regions and associated space charge fields, which are crucial in the functional performance of extrinsically doped semiconductor *p–n* junctions, was evident. Indeed, we do not think that such physics is relevant to the nature of domain-wall *p–n* junctions at all.

We note that LNO is the ideal system for these kinds of experiments, as the relative conductivities involved mean that source–drain currents are overwhelmingly driven along the walls, as opposed to through the domains (domains are many orders of magnitude more resistive than walls^2,3^).

All experiments were performed on commercially obtained, 500 nm thick, single-crystal ion-sliced LNO films (made and supplied by NANOLN). In the as-received state, the films were monodomain, with polarization pointing along the trigonal [001] direction (which lies parallel to the (21̅0) x-cut film surface). A 40 nm thick, ∼20 μm wide, Pt track was sputter-deposited onto the thin film surface, parallel to the polar axis; the tip of the atomic force microscope (AFM) was then used to machine away a thin strip of the metal (∼1–2 μm wide) to locally reveal the underlying LNO and to define an interelectrode gap and hence a coplanar capacitor structure (Figure 1b).

**Figure 1:**
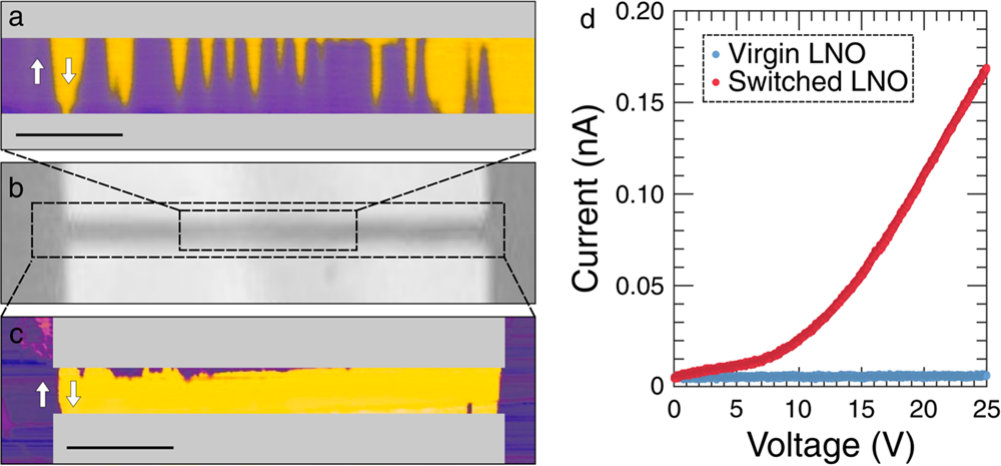
Switching behavior of domains between coplanar electrodes in x-cut lithium niobate (LNO). (a) Piezoresponse force microscopy (PFM) phase map of a typical domain microstructure in a partially poled state. (b) Optical image of the electrode geometry and interelectrode gap with regions of interest highlighted by dashed lines. (c) PFM phase map of the fully poled state. (d) *I*–*V* data corresponding to both the unswitched (blue) and fully switched (red) states, indicating percolating strongly conducting domain walls. The scale bar is 1.4 μm in (a) and 7 μm in (c), and the polar directions are indicated by the white arrows.

Polarization reversal was achieved by applying a voltage pulse across the electrodes, while simultaneously measuring the associated current (an indicator of the extent to which conducting walls straddled the interelectrode gap). Figure 1 illustrates the switching behavior. Lower voltage pulse magnitudes (between 80 and 100 V) induced a partially poled state, with a number of needle-like domains apparent (Figure 1a); higher voltages (>100 V) caused complete domain coalescence (Figure 1c) and strong conduction between the electrodes (Figure 1d). Details of the switching pulse and current response can be found in Figure S1 of the Supporting Information.

To establish domain morphology (as schematically represented in Figure 2a), we performed cross-sectional TEM imaging on focused ion-beam (FIB)-cut lamellae, oriented parallel to the polar axis and perpendicular to the film surface (lamellae parallel to the trigonal (010) plane), before and after switching. Figure 2b shows the locus of the domain wall associated with the fully switched domain state in this section. Its hemielliptical form unequivocally indicates head-to-head and tail-to-tail regions (which should be mediated by *n*- and *p*-type conduction, respectively^18^) as well as the *p–n* junction, located underneath the midpoint in the interelectrode gap. Such observations are consistent with previous research.^22,23^

**Figure 2:**
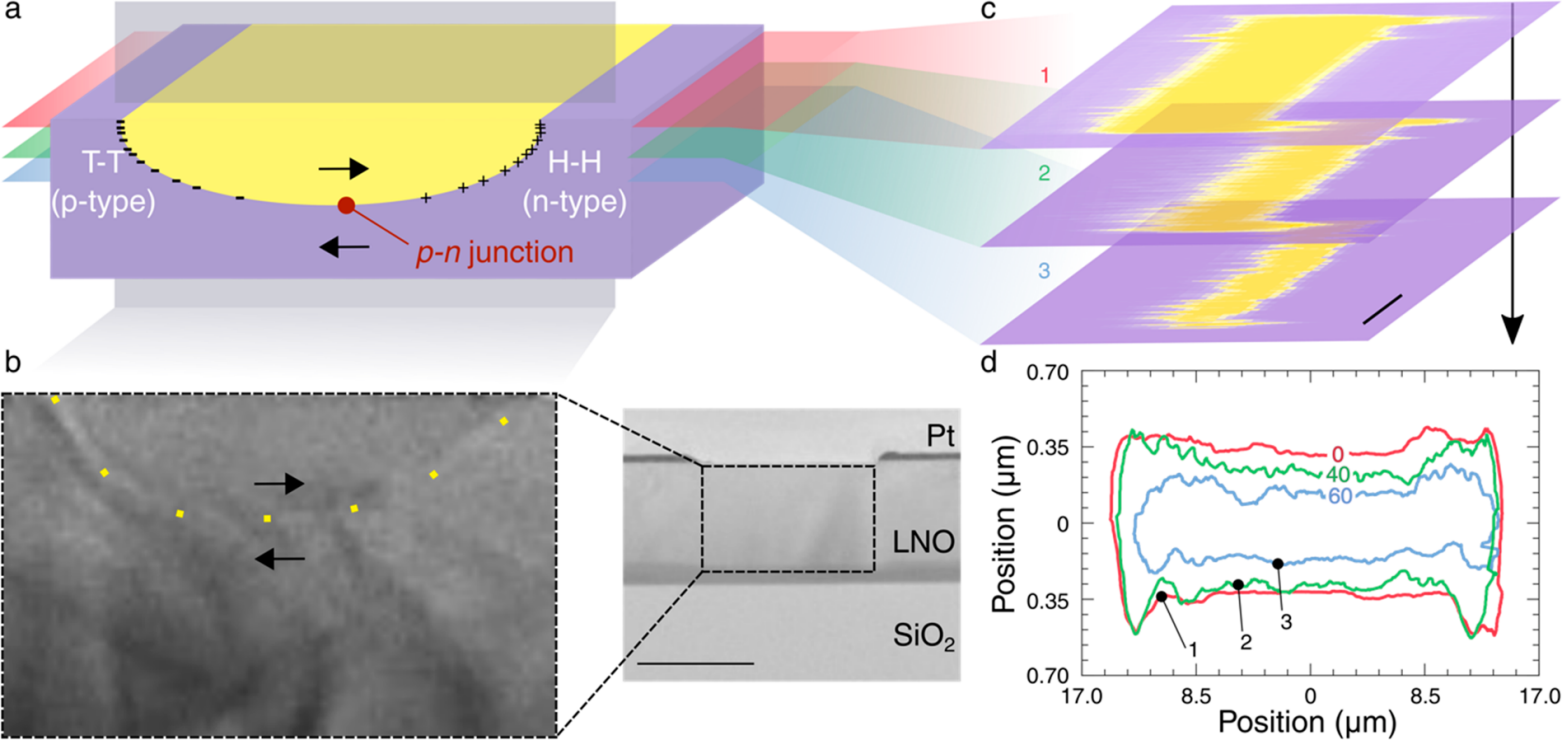
Domain microstructure in three dimensions. (a) Schematic illustration of the switched region of the lithium niobate (LNO). Head-to-head and tail-to-tail regions, with the associated bound charges, have been labeled, as has the *p–n* junction. (b) Cross-sectional transmission electron microscopy (TEM) image of the unswitched LNO (right), and a zoomed-in view of the fully poled region (left), which corresponds to the vertical plane in (a). The polar directions are indicated by the black arrows and the locus of the domain has been highlighted using yellow dots. (c) Tomographic piezoresponse force microscopy (TPFM) phase maps of the switched region between the electrodes (after they had been removed). The black arrow indicates the direction of increasing depth, from the film surface, and the numbers correspond to the domain-wall traces plotted in (d). Labels 0, 40, and 60 indicate the approximate acquisition depth below the surface in nm. The scale bar is 500 nm in (b) and 7 μm in (c).

Complementary morphological information parallel to the (21̅0) plane (parallel to the x-cut surface) was obtained using TPFM; this technique involves the application of significant force between the AFM probe and sample, such that layers of material are removed with each sequential area scan;^28−30^ piezoresponse force microscopy (PFM) information can be obtained during the milling process itself. Each scan hence generates a microstructural map from a different depth, such that the entire PFM data set can be used to reconstruct domain patterns in three dimensions. The TPFM phase images, stacked together in Figure 2c, show sections of the domain microstructure oriented parallel to the surface but at different depths below it. The switched domain appears to be approximately rectangular in all of these sections, but the aspect ratios of the rectangles change significantly with depth. This can be seen more explicitly by extracting the perimeter of the switched domain (the locus of the domain wall) and plotting it in the form of a subsurface contour map (Figure 2d). Taken together, the TPFM and TEM imaging suggest that the switched domain is hemielliptical in cross section and rectangular in planar section and that hence, in 3D, it is reasonably approximated as a hemielliptical column.

In the fully switched state, the equivalent circuit of the resistive elements across the interelectrode gap, for our experimental geometry, is that of domains and the domain wall acting electrically in parallel. The extremely insulating nature of the domains means that the domain contribution to the reciprocal of the parallel resistance is vanishingly small; at every point, the combined resistivity of the parallel components is thus completely dictated by the local resistivity of the wall. Hence, when currents are driven between the source and drain electrodes, the potential distribution developed at the surface of the thin film should reasonably reflect the potential present at each point along the subsurface domain wall.

We used high-voltage KPFM to capture this potential and the way it changed as different currents were driven along the wall and through the associated *p–n* junction. At relatively low driving currents, 2D maps (Figure 3a) showed a modest potential drop within the electrodes, while the vast majority was dropped across the interelectrode gap. As the current was increased, a much more noticeable potential drop developed in the grounded electrode. A more explicit illustration of these behaviors can be seen by taking the average of all potential profiles (Figure 3b) as a function of position along a vector perpendicular to the edges of the electrodes.

**Figure 3:**
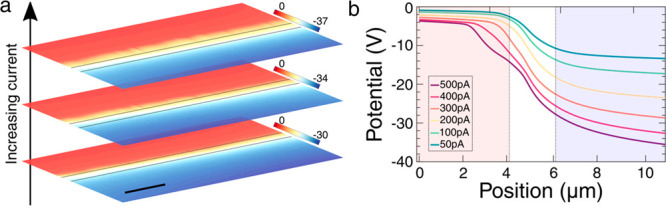
*In-operando* surface potential measurements. (a) Two-dimensional spatial maps of the measured surface potential as three different current magnitudes are driven along the curved conducting domain wall: 300 pA (bottom), 400 pA (middle) and 500 pA (top). The average position of the interelectrode gap across the width of the capacitor structure is marked by the black lines. The potential scale is in volts, and the scale bar represents 7 μm. (b) Average potential profiles, which have been cropped and centered around the interelectrode gap for each value of current. The shaded regions indicate the position of the grounded (red) and biased (blue) electrode. The two-dimensional potential maps for lower current magnitudes can be found in the Supporting Information.

By extracting gradients, the spatial distribution of the electric field could be realized, as a function of the current driven along the wall (Figure 4). A field peak around the middle of the interelectrode gap is clearly evident and was the dominant feature for all values of wall current. However, several other field peaks (or shoulders to the main central peak) occurred and, in general, developed more obviously as the current increased. One was spatially fixed at the edge of the earthed electrode. We suspect that this is a fringing field, commonly seen in capacitor structures (although we note that the other electrode does not seem to noticeably develop one). The other peak (or shoulder) was within the earthed electrode, progressing farther from the electrode edge, as the driving currents increased. Its origin is unclear, but we suspect that the increasing potential difference between the electrodes (needed to drive larger currents) causes the switched domain to expand and the junction between the electrode and tail-to-tail section of the domain wall to move. We speculate that the field peak might therefore be related to barrier resistance at the Pt–wall contact. However, we do not fully understand why and how this is measured “through” the electrode.

**Figure 4:**
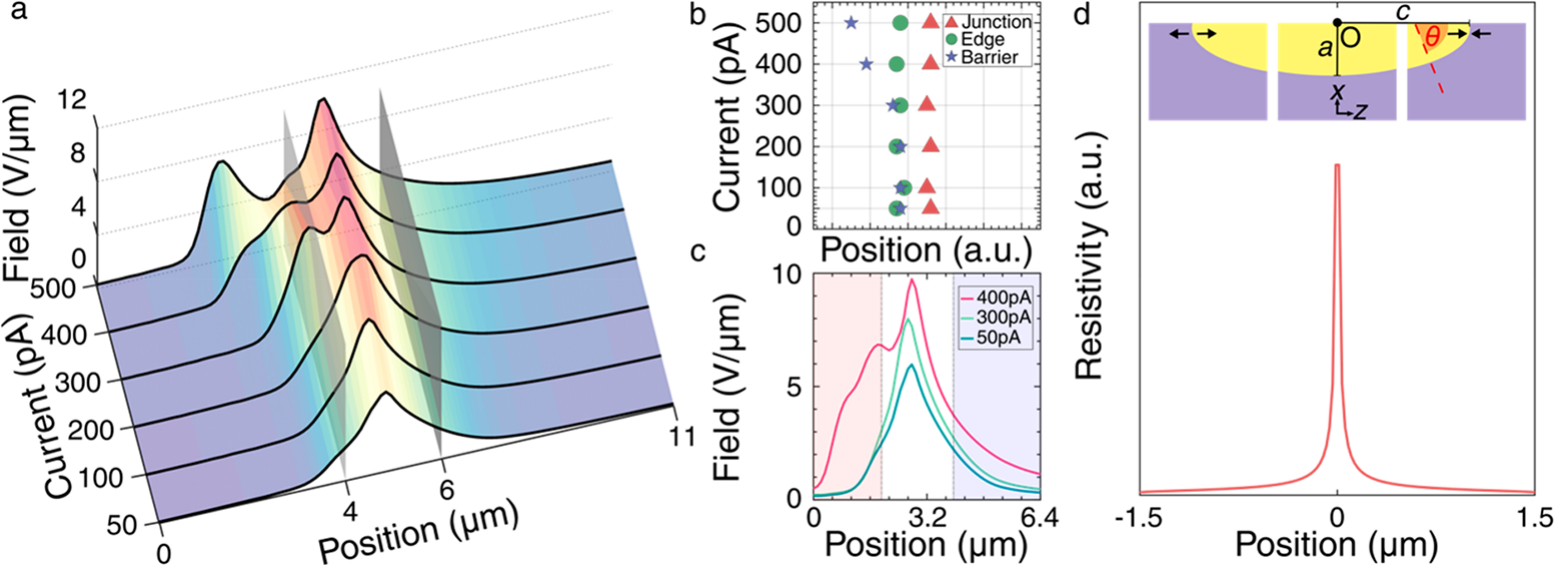
Interpretation of electric field profiles. (a) Three-dimensional plot of the electric field profiles obtained at different driving currents as a function of position along the polar axis. The vertical gray planes indicate the locations of the boundaries between the electrodes and the interelectrode gap. (b) Key peak or shoulder features apparent in the field data plotted as a function of position for each current (assigned as being associated with the *p–n* junction, electrode edge, and electrode–wall barrier). (c) Two-dimensional plot of the electric field vs position for a subset of data (50, 300, and 400 pA) with the electrodes indicated by the shaded regions as before. Additional field profiles are plotted in two dimensions in Figure S4. (d) Modeled resistivity profile for a domain wall based on the magnitude of the polar discontinuity at each point along the domain wall (depicted in the insets). Labeled are the origin, the semiminor “*a*” and semimajor “*c*” axes, the angle θ, and the polar directions.

In any case, at all bias levels and all driving currents, the domain wall *p–n* junction should remain firmly within the interelectrode gap, and hence we focus on the main field peak found there and what it might reveal about the physics of the junction.

Eng and co-workers have emphatically demonstrated that in LNO the spatial variation in domain wall conductivity is dependent on the local wall inclination angle, with respect to the polarization axis.^11^ Certainly, there is an expectation that this angle will control the charge density needed to screen the polar discontinuity at each point; moreover, the screening charge density should be reflected in the carrier density associated with transport.^9,10,31^ However, carrier mobilities also seem to show angular dependence: those determined for LNO walls with relatively shallow inclinations with respect to the polar axis seem to be dramatically lower than those seen in walls that are more steeply inclined.^32^ Evidently, carrier density and mobility variations occur in the same sense and thus act in concert to either enhance or diminish local wall conductivity.

In the curved domain walls examined herein, inclination angles, with respect to the polar axis, change continuously from the source to drain. Given the information on the domain-wall morphology revealed by TEM and TPFM, we have considered the domain-wall locus, in two dimensions, to be that of a hemiellipse with origin on the thin film surface and at the midpoint of the interelectrode gap (Figure 4d):
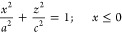
1Here, the *z*-axis is parallel to the polarization vector (parallel to the trigonal [001]) and the *x*-axis is perpendicular to the LNO film surface (parallel to the trigonal [100]). *a* and *c* are the magnitudes of the semiminor and semimajor axes, respectively, in the domain-wall ellipse (in our case, *a* ∼ 150 nm and *c* ∼ 1.5 μm). The component of the unit vector normal to this elliptical locus, resolved parallel to the *z*-axis, scales with the magnitude of the local polar divergence (∇·*P*) at each point on the domain wall and can be given as a function of *z*:
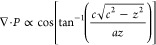
2This function should also scale with the local conductivity, as discussed above. The spatially varying field needed to drive the same current through every part of the elliptical pathway of the domain wall (considering that all local regions along the hemielliptical wall section act electrically in series) will scale directly with the local wall resistivity and hence with the inverse of the function given in eq 2. This inverse function is plotted in Figure 4d and shows an obvious peak at the midpoint (in principle, this is a singularity), where the tangent to the hemielliptical domain wall is parallel to [001]. This is the point at which the *p–n* junction occurs and where by definition no polar discontinuity exists. The similarities between the experimentally determined function (in Figure 4b) and that modeled (in Figure 4d) show that the measured field peak, associated with the *p–n* junction, can be broadly understood by solely considering expectations for the variations in resistivity along the wall.

The *in-operando* fields developed at conventional *p–n* junctions, between differently doped extrinsic semiconductors, appear similar^33^ but instead originate from a high resistance depletion region, resulting from an equilibrium local redistribution of electrons and holes. The formation of this depletion region is entirely dependent on relatively high energy electrons (in the *n*-type material, within the bandgap and close to the bottom of the conduction band) being spatially adjacent to relatively low-energy acceptor states (in the *p*-type material, within the bandgap and close to the top of the valence band) and the associated differences in the Fermi levels of the two materials when not in contact. In the LNO domain wall, the Fermi levels for both the *n*- and *p*-type sections (even if spatially separated) are determined by their common contact with the surrounding bulk domains.^5,34^ There can be no Fermi level differences between them and hence no drive for carrier redistribution, beyond that associated with entropy, when *n*- and *p*-type walls meet. Thus, not only can the measured field peak at the domain-wall *p–n* junction be rationalized completely by expected resistivity variations along the wall (resulting from local changes in the magnitude of the divergence in polarization) but also the depletion region physics (a defining characteristic of conventional *p–n* junctions in extrinsic semiconductors)cannot occur. Domain-wall *p–n* junctions are thus very different from conventional *p–n* junctions and cannot produce the diode behavior that is the central motivation for their integration into semiconductor devices.

We note that we have done preliminary *in situ* TEM imaging which shows that, as source–drain bias fields and currents increase, the head-to-head and tail-to-tail sections of the domain wall appear to increase their local angles of inclination, with respect to the polarization axis. This has the effect of locally increasing domain-wall conductivity and “sharpening” the region over which the *p–n* junction occurs, making it more spatially defined than is apparent in the TPFM and TEM images presented in Figure 2. This has no dramatic effect on the form of the local field measurements, around the *p–n* junction region, and so there is no evidence for a change in response, with increased spatial confinement of the junction.

In summary, we have established the morphology of curved domain walls that contain *p–n* junctions, in locally switched x-cut lithium niobate, and measured the spatial distributions in potential that develop, when currents are driven along them. A modeled resistivity profile, informed solely by the local divergence of polarization along the wall, is sufficient to explain the main feature seen in the electric field distribution. Our data therefore imply that abutting *p*- and *n*-type domain walls behave fundamentally differently to conventional extrinsically doped semiconductor *p–n* junctions. Crucially, domain-wall *p–n* junctions do not develop carrier depletion zones; Fermi level differences between *p*- and *n*-type domain walls do not exist because of their unavoidable contact with a common matrix (the surrounding LNO). Such findings have implications for the development of domain-wall-based nanoelectronic devices, as they show that simply joining *p*- and *n*-type domain walls together will not be enough to generate the device functionality conventionally expected.

## Experimental Methods

### Sample Preparation

We used commercially obtained 500 nm thick, ion-sliced x-cut lithium niobate films, bonded onto a SiO_2_/LNO substrate (from NanoLN). To create the capacitor structure, copper TEM grids were used as the hard masks. A 25 μm × 2 mm, 40 nm thick Pt bar electrode was deposited through the hard masks, parallel to the polar axis ([001]), by magnetron sputtering. A diamond-coated AFM probe with a spring constant of 80 N/m (supplied by NanoWorld) was used, under high set points (force of ∼1 μN), to remove an area of the Pt and create an interelectrode gap approximately 1–2 μm wide. All scanning probe experiments were performed on an MFP-3D Infinity AFM system. Discharging of the capacitor plates by connecting to ground was performed throughout processing to minimize the effects of electrostatic discharge (ESD).

### Domain Switching and Characterization

The AFM system was used in conjunction with a High Voltage Option (ORCA mode) to apply the switching pulse and record the *I*–*V* behavior. A standard Pt/Ir-coated Si probe (Nanosensors, PPP-EFM) was used to apply the voltage and measure current. Lateral PFM imaging was performed at resonance with a standard tip, at a frequency of ∼650 kHz and an AC bias of 2 V. Tomographic PFM imaging was performed with diamond-coated probes and at high set points (force of ∼1 μN).

### Transmission Electron Microscopy

Bright-field TEM images were acquired on a Thermofisher Talos F200X instrument operated at 200 kV.

### High-Voltage Kelvin Probe Force Microscopy

Currents driven across domain walls were supplied using an external Keithley 237 source-measure unit. The potential mapping across electrodes was undertaken using standard Pt/Ir-coated Si probes (Nanosensors, PPP-EFM with free resonance of ∼70 kHz) in high-voltage KPFM mode. A high-voltage (HV) module, custom designed for the MFP Infinity system, was used, around which the HV-KPFM measurement mode is designed. The HV module facilitates precise AC+DC signals between −150 and +150 V, thereby expanding the range of the KPFM measurements beyond the conventional ±10 V range. Such measurements are not offered as a standard option and needed software/hardware customization so that the controller can send a large (>10 V) DC voltage to the tip, in the interleave mode, to nullify oscillations and thereby determine the potential. During scanning in the interleave pass, the appropriate mix of AC and DC voltages is directly routed to the tip through the AFM software. The software control panel has been customized such that the nullifying voltage can be directly recorded at each pixel in a precise manner. The HVKPFM mode was tested for applied voltages on a deposited gold pad and found to be able to measure voltages accurately (within 3%) and precisely (noise floor <0.2 V).

## Data Availability

The data underlying this study are openly available in the PURE research database at https://doi.org/10.17034/7b295b79-6713-47bf-a850-02dee05907e8.

## Supporting Information Available

Four figures illustrating: the current between source and drain electrodes developed during switching (Figure S1); raw and smoothed data, associated with the domain-wall locus, taken using tomographic piezoresponse force microscopy, without any driving current (Figure S2); additional high-voltage Kelvin probe microscopy maps at lower driving domain-wall currents than those in the main text (Figure S3); and plots of the electric field distribution needed to drive all the domain-wall currents investigated (Figure S4) (PDF)
