# Supplementary material for: Ferroelectric Domain Wall p–n Junctions
Source: Nano Lett. 2023 Nov 10;23(22):10360–6. doi: 10.1021/acs.nanolett.3c02966 (PMC10683062; doi:10.1021/acs.nanolett.3c02966)
Supplement: Supplementary file 1 — nl3c02966_si_001.pdf [file nl3c02966_si_001.pdf]

Supporting Information

Ferroelectric Domain Wall p-n Junctions

Jesi R. Maguire[1#], Conor J. McCluskey[1], Kristina M. Holsgrove[1], Ahmet Suna[1,2], Amit Kumar[1], Raymond G. P. McQuaid[1] and J. Marty Gregg[1*]

[1] School of Mathematics and Physics, Queen's University Belfast, Belfast, BT7 1NN, U.K.

[2] Analog Devices Ltd, Dower House, London Rd, Newbury, RG14 1LA, U.K.

Corresponding author emails: #jmaguire62@qub.ac.uk, *m.gregg@qub.ac.uk

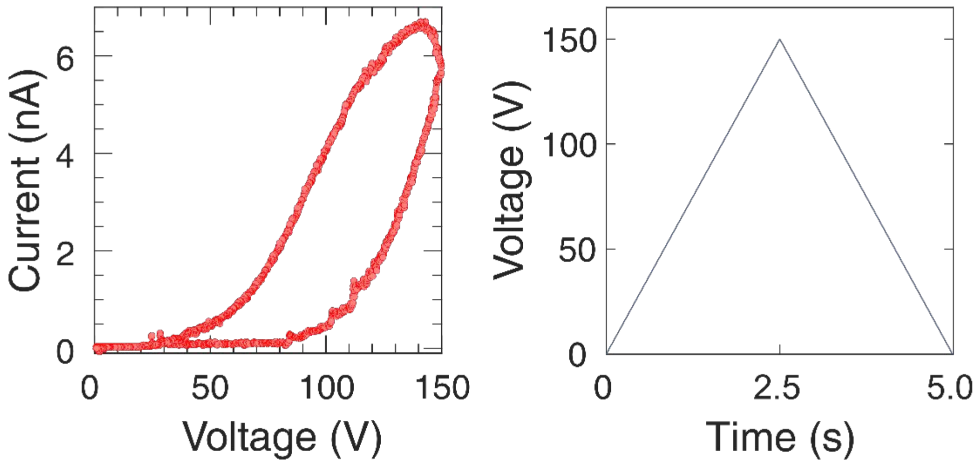

Figure S1: Response of the LNO, in terms of the domain wall current driven between source and drain electrodes (left) associated switching pulse (right). The voltage was swept from 0 → 150 V and back to 0 V using a High Voltage Option (ORCA™ mode) in an Asylum Research MFP-3D Infinity AFM system. The voltage at which switching current is first observed (where significant domain wall percolation is induced) appears to be approximately 80 V. The current value on the ramp down is higher than it is for the same voltage on the ramp up, as the domain wall was created during the ramp up phase of the pulse (and was in-place for the ramp down).

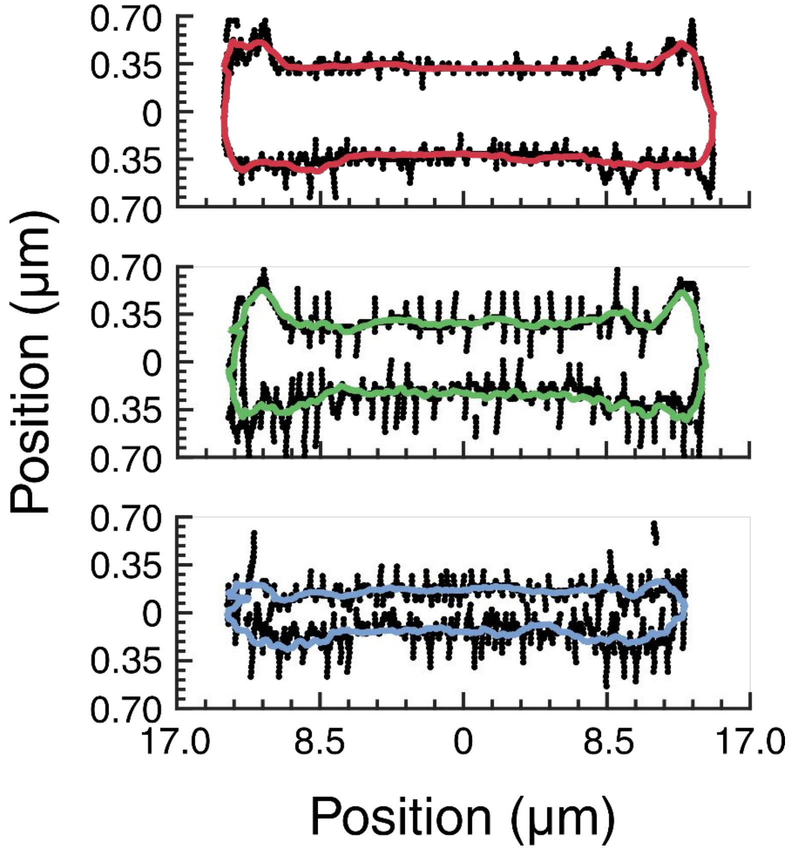

Figure S2: Smoothed representations of the domain wall loci at different depths below the surface of the x-cut lithium niobate within the interelectrode gap (coloured lines); these are plotted on top of the raw data for the domain walls (in black). The domain wall loci were extracted from tomographic piezoresponse force microscopy (TPFM) images by identifying maxima in the spatial gradient of the phase. The data was smoothed to eliminate the noise spikes which become more pronounced as a function of increasing depth (top to bottom).

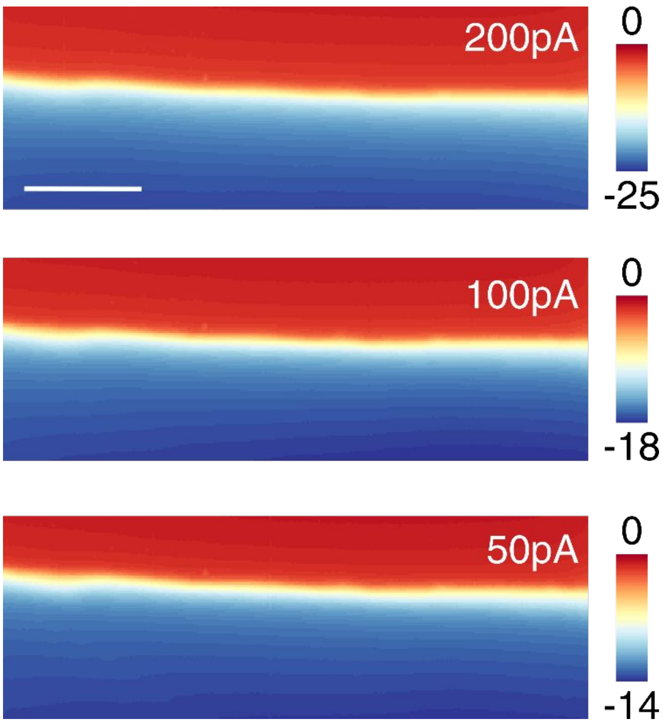

Figure S3: Two dimensional potential maps measured as a function of current values lower than those shown in the main text: 200pA (top), 100pA (middle) and 50pA (bottom). The colour scale is measured in volts and the scale bar applies to all three maps and measures 7 μm. Most of the potential, at these current values, is dropped across the interelectrode gap.

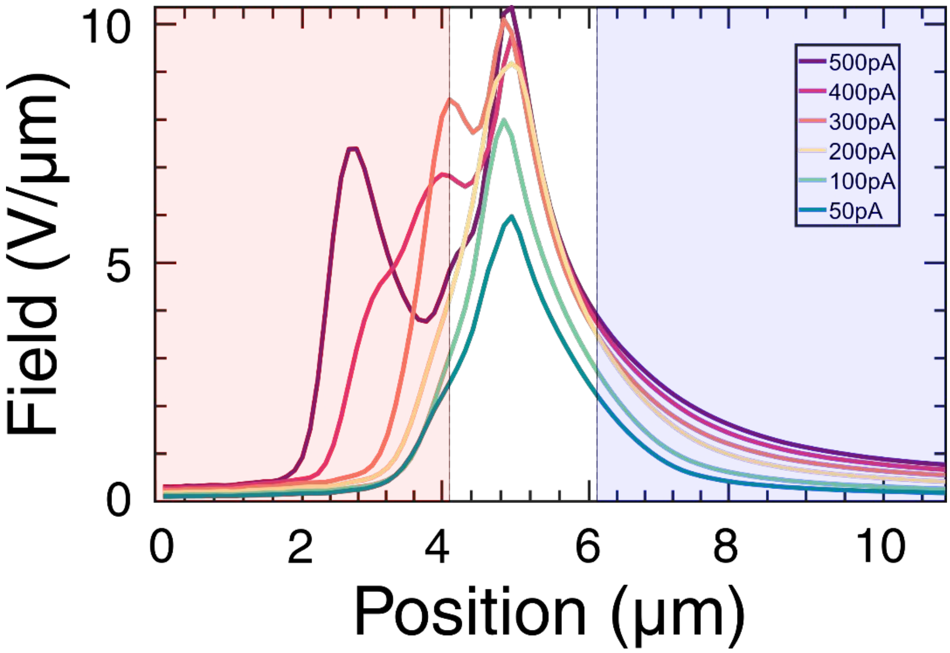

Figure S4: Two-dimensional plots of the electric field vs. position for a larger number of magnitudes of driven current than presented in the main article. The positions of the electrodes are indicated by the shaded regions. The interelectrode gap is unshaded.
